# Optimizing the generation of mature bone marrow-derived dendritic cells in vitro: a factorial study design

**DOI:** 10.1186/s43141-023-00597-4

**Published:** 2023-11-29

**Authors:** Najla Alotaibi, Alia Aldahlawi, Kawther Zaher, Fatemah Basingab, Jehan Alrahimi

**Affiliations:** 1 Department of Biological Sciences, Faculty of Sciences, King Abdulaziz University, Jeddah, Saudi Arabia; 2 Immunology Unit, King Fahad Medical Research Center, King Abdulaziz University, Jeddah, Saudi Arabia; 3 College of Health, Oregon State University, Corvallis, OR USA

**Keywords:** Dendritic cells, Factorial design, Optimization, GM-CSF, IL4, DoE

## Abstract

**Background:**

Factorial design is a simple, yet elegant method to investigate the effect of multiple factors and their interaction on a specific response simultaneously. Hence, this type of study design reaches the best optimization conditions of a process. Although the interaction between the variables is widely prevalent in cell culture procedures, factorial design *per se* is infrequently utilized in improving cell culture output. Therefore, we aim to optimize the experimental conditions for generating mature bone marrow-derived dendritic cells (BMDCs). Two different variables were investigated, including the concentrations of the inducing factors and the starting density of the bone marrow mononuclear cells. In the current study, we utilized the design of experiments (DoE), a statistical approach, to systematically assess the impact of factors with varying levels on cell culture outcomes. Herein, we apply a two-factor, two-level (2^2^) factorial experiment resulting in four conditions that are run in triplicate. The two variables investigated here are cytokines combinations with two levels, granulocyte–macrophage colony-stimulating factor (GM-CSF) alone or with interleukin-4 (IL4). The other parameter is cell density with two different concentrations, 2 × 10^6^ and 4 × 10^6^ cells/mL. Then, we measured cell viability using the trypan blue exclusion method, and a flow cytometer was used to detect the BMDCs expressing the markers FITC-CD80, CD86, CD83, and CD14. BMDC marker expression levels were calculated using arbitrary units (AU) of the mean fluorescence intensity (MFI).

**Results:**

The current study showed that the highest total viable cells and cells yield obtained were in cell group seeded at 2 × 10^6^ cells/mL and treated with GM-CSF and IL-4. Importantly, the expression of the co-stimulatory molecules CD83 and CD80/CD86 were statistically significant for cell density of 2 × 10^6^ cells/mL (*P* < 0.01, two-way ANOVA). Bone marrow mononuclear cells seeded at 4 × 10^6^ in the presence of the cytokine mix less efficiently differentiated and matured into BMDCs. Statistical analysis via two-way ANOVA revealed an interaction between cell density and cytokine combinations.

**Conclusion:**

The analysis of this study indicates a substantial interaction between cytokines combinations and cell densities on BMDC maturation. However, higher cell density is not associated with optimizing DC maturation. Notably, applying DoE in bioprocess designs increases experimental efficacy and reliability while minimizing experiments, time, and process costs.

## Background

Dendritic cells (DCs) are immune cells that link the innate and adaptive immune systems. They are derived from bone marrow (BM) precursors and patrolling the blood. Functionally and morphologically, DCs consist of a heterogeneous population with different phenotypic subsets and locations [1]. They are abundant in epithelia and lymphoid organs, such as the skin [2], blood [3], lymph node [1], lungs [4], stomach [5], and intestines [6]. Crucially, DCs have a role in activating cytotoxic T cells and therefore have been extensively used in developing cancer immunotherapies [7]. DC maturation is triggered after immature DCs are exposed to pathogen-associated molecular patterns (PAMPs) [8, 9]. They have been recognized for their essential role as professional antigen-presenting cells (APCs). This process is represented by recognizing the antigen and processing it into small peptides, then presenting it on their major histocompatibility molecule (MHC) to activate cytotoxic T cells [10, 11]. As a result, DCs will undergo morphological changes including migrating to lymphatic organs, expressing co-stimulatory molecules, and secreting cytokines [9].

Given their potent ability to process and present antigens, DCs hold a promising treatment for multiple advanced diseases including cancer. Moreover, DC-based therapies are used for other medical purposes, such as preventing transplant rejection [10]. Furthermore, several studies have shed light on combining DC-based vaccines with conventional therapies like chemotherapy to increase their efficacy [11–13], which helped the development of several DC-based therapeutic vaccines [14].

Due to the higher reachability and accessibility, BM became one of the primary sources of DC precursors. In in vitro studies, mature BMDCs are mainly generated by culturing BM monocyte progenitor with GM-CSF alone or combined with IL4 [8]. GM-CSF is essential and proven to maximize pure DCs [9]. Besides, cytokines like IL-4 enhance DC maturation from CD34^+^ and CD14^+^ precursors [8, 9]. The BM of BALB/c mice was used to obtain hematopoietic precursor cells, whereas early washings removed non-DCs [9, 10].

Commonly, the maturation of DCs is evaluated by flow cytometric analysis of associated markers. The markers were expressed on the surface of the immune cells and each marker was investigated individually [15]. In DC flow cytometry analysis, the common markers include the monocyte surface marker CD14, which decreases when monocytes are differentiating into macrophages or DCs besides the common DC maturation marker CD83 and costimulatory molecules CD80/CD86 [16, 17].

For optimal T activation, DCs need to deliver two signals: signal 1, where DCs are effectively presenting peptides to the T cell receptor (TCR) via the MHC molecule. Plus, DC costimulatory molecules as signal 2, where the B7 family (CD80/CD86) is engaged to the CD28 receptor on the T cell surface [12, 17]. Engagement of CD28 on naïve T cells by either B7-1 or B7-2 ligands on APCs provides a potent costimulatory signal which resulted in induction of IL-2 transcription, expression of CD25, and entry into the cell cycle. CD28 engagement also confers critical survival signals to activated T cells [17].

Interestingly, there remains a noticeable gap in systematic analyses investigating the influence of parameter levels and their interactions on culture performance. While the existing body of literature acknowledges the utility of factorial design in probing interactions, its application remains limited in cell culture research. We take a pioneering step by not only filling this gap but also contributing novel insights into the application of factorial design to optimize DC maturation, representing an innovative advancement in cell culture methodologies. This novel approach resonates with a similar mode of factorial study design that has emerged in recent works, wherein the application of cell-biomaterial full factorial design yielded valuable insights into optimizing cell density efficiency [18, 19], primary culture conditions, cytokine and serum doses on stem cells [20], solid lipid nanoparticles [21], cell suspension culture media for maximizing antibody production [22], tissue engineering [18, 23], and various biotechnological studies [24, 25]. Our contribution to this field is especially pertinent given the critical role of generating mature DCs in DC-based immunotherapies. While optimization conditions for mature DCs in cell culture are underexplored, applying factorial design has proven effective in enhancing our understanding and knowledge to optimize cell maturation within this context.

The aim of the present work is to use a DoE statistical approach to investigate the main effects of culture variables and their interaction in the optimization of the optimization of generation of mature BMDCs. Thus, this statistical approach was applied to a two-factor, two-level (2^2^) factorial experiment. The cytokine combinations are granulocyte–macrophage colony-stimulating factor (GM-CSF) alone and GM-CSF plus interleukin-4 (IL4). While the cell densities are 2 × 10^6^ and 4 × 10^6^ cells/mL. Accordingly, BMDCs were generated from murine BM and differentiated using appropriate cytokines and seeded at specific cell densities according to the study design. The yield and viability of BMDCs were assessed by Trypan blue exclusion, and phenotypic characterization of mature BMDCs was evaluated by flow cytometric analysis. By applying the statistical approach to the design of the experiment, we could contribute to improving cell culture procedures by understanding the main influence of desired factors and their interaction on cell culture.

## Methods

### Reagent and antibodies

Roswell Park Memorial Institute medium (RPMI), phosphate-buffered saline (PBS), heat-inactivated fetal bovine serum (FBS), Trypan blue exclusion assay, and lipopolysaccharide (LPS) were purchased from Sigma Aldrich, MO, USA. Penicillin/Streptomycin 10,000 unit/mL and l-glutamine were purchased from Thermo Fisher Scientific, USA, Mouse recombinant IL4 and FITC-antibodies (CD80, CD86, CD83, and CD14) were purchased from BioLegend® (London, UK). Mouse recombinant GM-CSF was purchased from R&D systems® (MO, USA).

### Animal

Female BALB/c (6 to 8 weeks, weight = 19.48 ± 0.21) were obtained from the Animal House and bred in specific pathogen-free conditions. The mice were maintained in the animal house with unrestricted access to water and a balanced diet. They were kept under laboratory conditions with a temperature of 22 °C (± 2), 40–60% humidity, and illuminations with a 12-h light–dark cycle in wire-bottomed cages.

### Factorial study design to optimize BMDC maturation

Full factorial experimental design (*i*^*k*^) is an experimental design that includes all possible combinations of *k* factors at two or more levels [26, 27]. Herein, a combination of levels for each factor included in the study will be run separately and will result in *i*^*k*^ set of experimental runs. In this work, optimal conditions of cytokine combinations and cell density to enhance BMDC maturation were determined using a factorial design (2 levels, 2 factors).

The response to optimizing BMDC maturation (Y) was studied using independent variables of cytokine combinations (A) and cell density (B). The cytokine combination was GM-CSF and GM-CSF plus IL4, while the cell density levels were 2 × 10^6^ and 4 × 10^6^ cells/mL. The experimental runs were determined according to the requirements of a full factorial design using the following equation [28, 29]:

$$Experiments\ number=Level{s}^{Factors}$$

Full factorial experimental runs are constructed here by two-factor two-levels (2^2^), resulting in a total of four conditions with triplicate; therefore, there will be 12 experimental runs.

### Generating mice bone marrow-derived dendritic cells

Generating of BMDCs was performed according to a paper described by Inaba, 1992, with a slight modification [30, 31]. Briefly, 6- to 8-week BALB/c mice (*n* = 2) were euthanized according to guidelines and regulations of local animal care by inhalational anesthetic isoflurane, and death was confirmed by cervical dislocation. The skin was removed, then the femurs were separated by cutting the connection point with a scissor. Muscles and tissues around the femur and tibia were removed under sterile conditions. The harvested bones were placed into a 100-mm non-tissue culture-treated plate filled with 70% ethanol for 2 min and then transferred to a culture-treated plate containing RPMI-1640 medium. After cutting both ends of the femur with scissors, a needle of a 1-mL syringe filled with complete cold RPMI-1640 was inserted into the bone cavity to flush out the marrow from the bone into the petri dish containing complete RPMI-1640. Then, the BM cell solution was pipetted to form a single cell suspension and transferred to a 15-mL conical tube by cell strainer to remove any residuals, then centrifuged at 300 × g for 10 min at 4 °C to pellet the cells. Briefly, the BM collected cell suspension was washed with PBS twice at 300 × g for 10 min at 4 °C. Cells were resuspended in 5–10 mL of ACK (ammonium-chloride-potassium) lysing buffer and were incubated for 3–5 min at room temperature to lyse red blood cells. Five to 10 mL of complete RPMI-1640 media was added, and cells were washed twice.

To apply factorial design, cells were counted using a hemocytometer and adjusted to two main densities equal to 2 × 10^6^ cells/mL or 4 × 10^6^ cells/mL. Cells were then seeded in 6-well plates in complete RPMI-1640 media (i.e., 3 mL/well) supplemented by cytokine combinations of recombinant murine GM-CSF and recombinant murine IL4 to a final concentration of 20 ng/mL and 10 ng/mL, respectively, while other cells were cultured in complete media supplemented by only a recombinant murine GM-CSF (Table 1). Cells were cultured at 37ºC in an incubator containing 5% CO_2_. On day 3 and day 5, half of the media was removed, and new media supplemented with appropriate cytokines was added. On day 6, BMDCs were activated by adding 1 μg/mL of LPS for 24 h before cells were collected [32].

**Table 1:** The levels of two independent variables used in (2^2^) factorial design

Independent variables	Model symbol	Levels	
		**Low**	**High**
**Cytokine combination**	A	GM-CSF^a^	GM-CSF^a^ & IL4^b^
**Cell density**	B	2 × 10^6^ cells/mL	4 × 10^6^ cells/mL

*GM-CSF* granulocyte–macrophage colony-stimulating factor, *IL-4* interleukin-4

^a^GM-CSF was 20ng/mL

^b^IL4 was 10ng/mL

### Cells yield and viability

At day 7, BMDCs were collected, and a Trypan blue exclusion assay was used to assess the yield and viability of BMDCs as described previously. Briefly, viable DCs were assessed by counting elongated, irregular with numerous and long cytoplasmic projection cells. The percentages of cells yield were estimated using the formula: yield (%) = BMDCs/total cells × 100.

### Phenotype analysis of mature bone marrow-derived dendritic cell

Phenotype properties of maturation status were investigated by calculating the MFI of mature BMDC surface markers. BMDCs collected on day 7 were washed twice with flow cytometry staining buffer (FACS; PBS + 1% FBS) and incubated with the following antibodies; CD80, CD86, CD83, and CD14 for 30min at 4°C. Cells were washed twice with FACS to remove excessive antibodies [22, 33]. Stained and unstained cells were acquired using flow cytometry (Beckman Coulter Life Science, USA) using 5000 events. Data was analyzed using FCS Express 7 (De Novo software, CA, USA). A factorial equation was performed to estimate the response as follows [34]:

$$Y={\beta }_{0}+ {\beta }_{1}A+ {\beta }_{2}B+ {\beta }_{12}AB+e$$

where *Y* is the estimated response, ${\beta }_{0}$ is the intercept value, ${\beta }_{1}$ and ${\beta }_{2}$ are the linear coefficient, ${\beta }_{12}$ is the factorial coefficient, A and B are the independent variables, and *e* is the residual error.

### Statistical analysis

The effect of different levels of the two independent variables on the response was evaluated using analysis of variance (ANOVA). Regression models of parameters (factorial equations) and their interactions and the coefficient of determination (*R*^2^) were estimated. All experiments were triplicated, and a *P* value of ≤ 0.05 was considered significant. Data analysis was performed using Rstudio (version 1.4.1106) (R Foundation for Statistical Computing, Vienna, Austria). The graphics were made using “ggpubr” and “grid” packages.

## Results

### Yield and viability of BMDCs

**Fig. 1:**
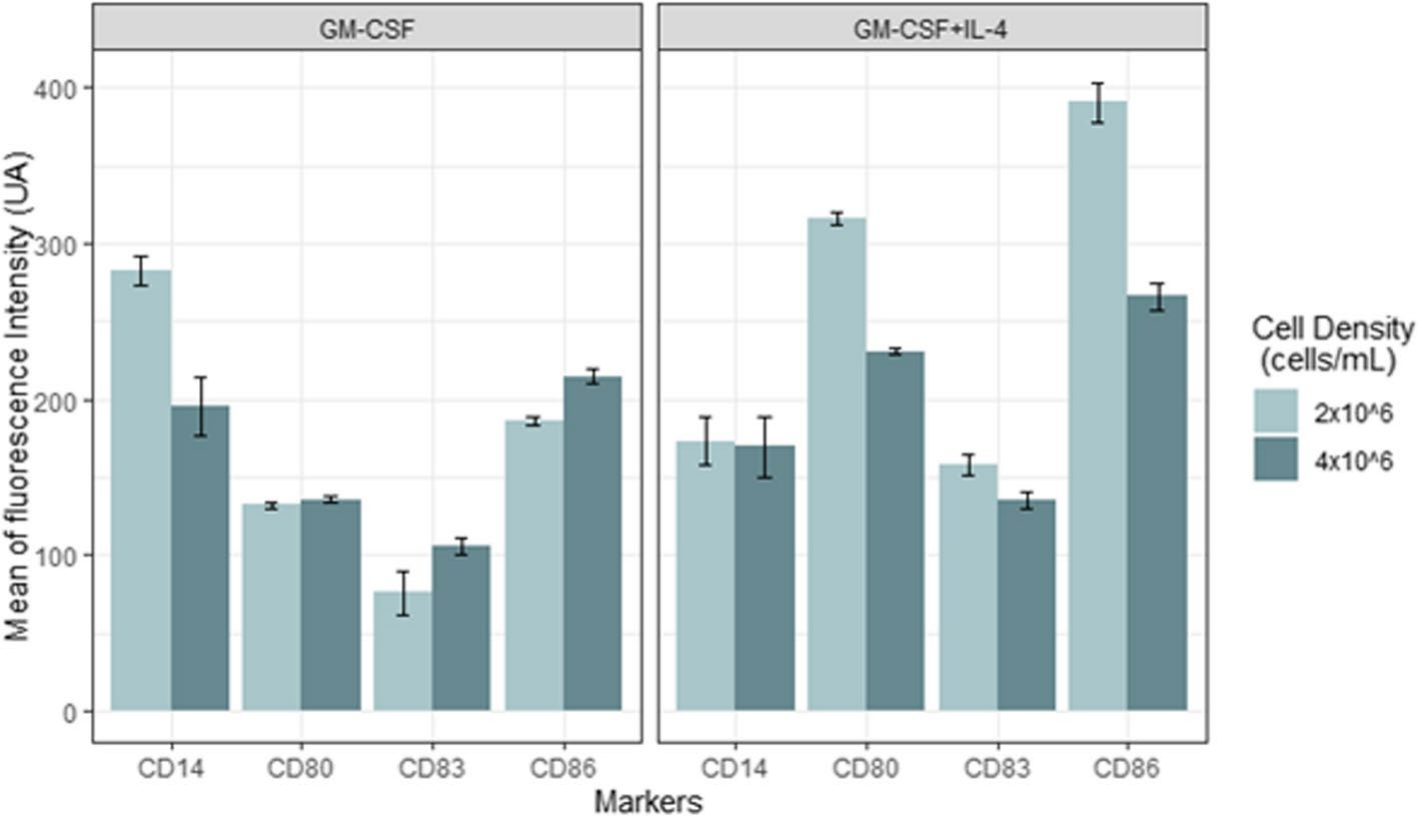
Phenotypic analysis of dendritic cell markers using flow cytometry. Barplot presents mean fluorescence intensity (MFI) of the cell’s markers based on cell density and cytokine combinations

The highest total viable cells obtained was 95.3% ± 1.4%, and cells yield were 6–6.5 × 10^7^ cells/mL in BMDCs that were cultured in the presence of both cytokines and seeded at 2 × 10^6^ cells/mL (Table 2) (Fig. 1).

**Table 2:** Comparison of dendritic cells yield and total viable cells

	**GM-CSF & IL4**		**GM-CSF**		^*^P
	**2** × **10**^**6**^	**4** × **10**^**6**^	**2** × **10**^**6**^	**4** × **10**^**6**^	
**Yield (cells/ml)**	6–6.5 × 10^7^	4.5–5 × 10^6^	4 × 10^6^	5–6 × 10^6^	0.465
**Viable cells (%)**	95.3 ± 1.4	91.5 ± 0.6	78.4 ± 3.4	83.1 ± 1.7	0.027

*GM-CSF* granulocyte–macrophage colony-stimulating factor, *IL-4* interleukin-4

^*^P value was based on two-way ANOVA

### Generating and phenotyping of mature BMDCs

**Fig. 2:**
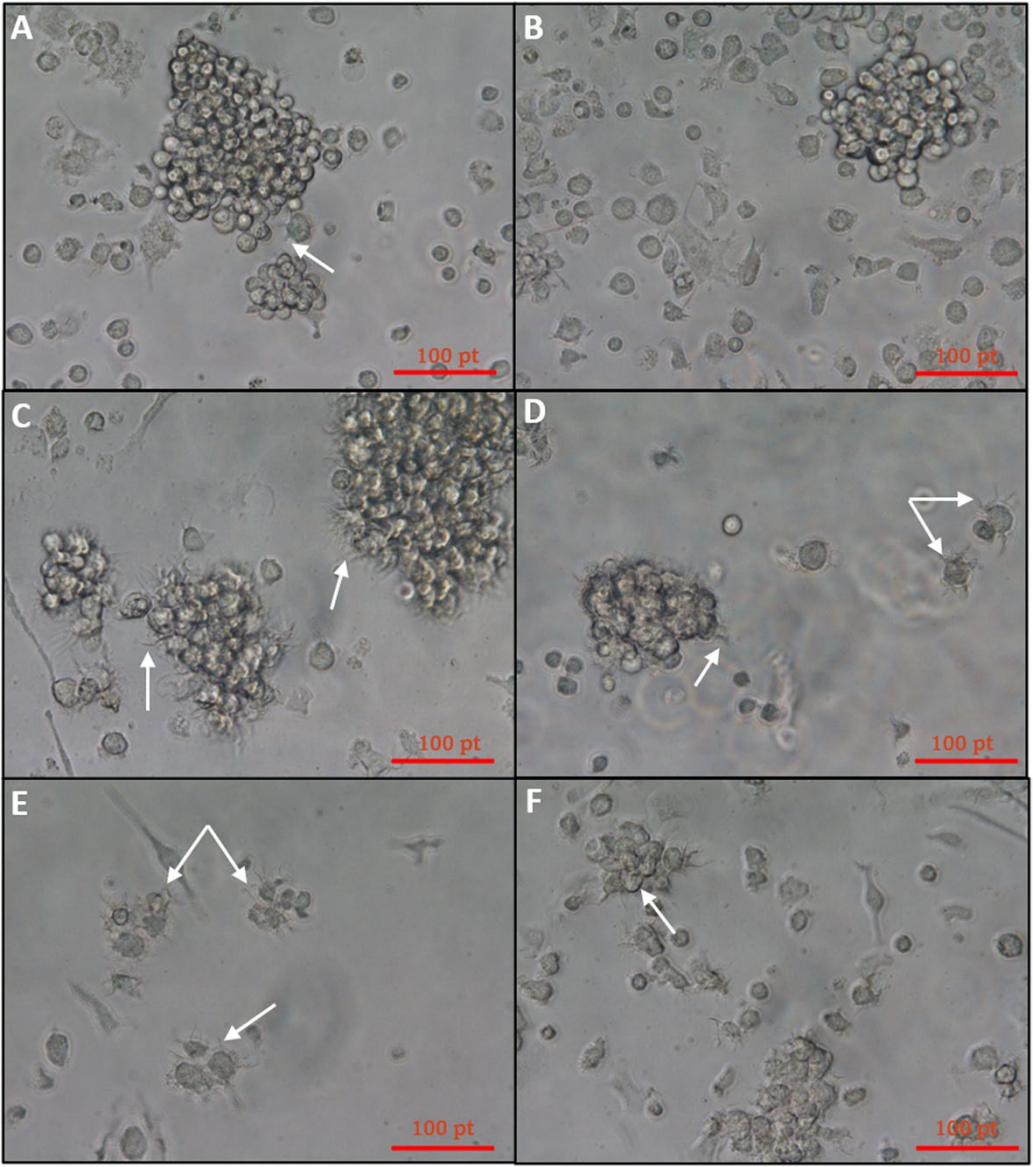
Morphological changes of mature BMDCs. **A**, **B** Unstimulated DCs (controls) were cultured for 7 days without cytokines at cell densities of 4 × 10^6^ and 2 × 10^6^ cells/mL, respectively, and appeared rounded in shape with no cytoplasmic projections. **C** DCs were cultured in the presence of GM-CSF and IL4 and seeded with a density of 2 × 10^6^ cells/mL. **D** DCs were cultured in the presence of GM-CSF and IL4 and seeded with a density of 4 × 10^6^ cells/mL while in **E** DCs were cultured in the lack of IL4 and with a density of 2 × 10^6^ cells/mL (the weakest cell appearance among other combination) and lastly in **F** DCs were also supplemented with only GM-CSF and lack of IL4 with a density of 4 × 10^6^ cells/mL. Pictures were taken with an inverted microscope (magnifications were 40 ×); arrows indicate dendrons of DCs

**Fig. 3:**
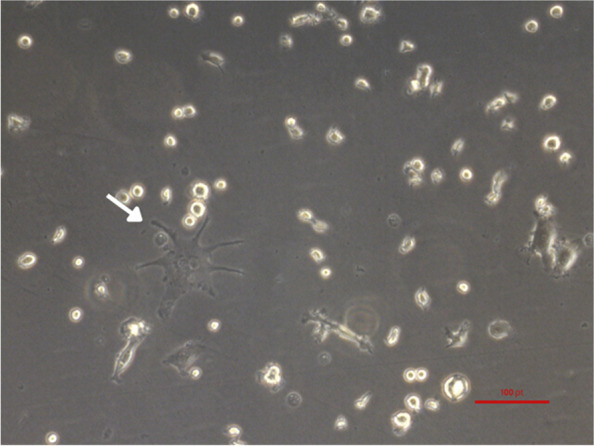
BMDC of optimized conditions. BMDCs supplemented GM-CSF and IL4 and seeded with a cell density of 2 × 10^6^ cells/mL. The picture was taken with an inverted microscope

**Fig. 4:**
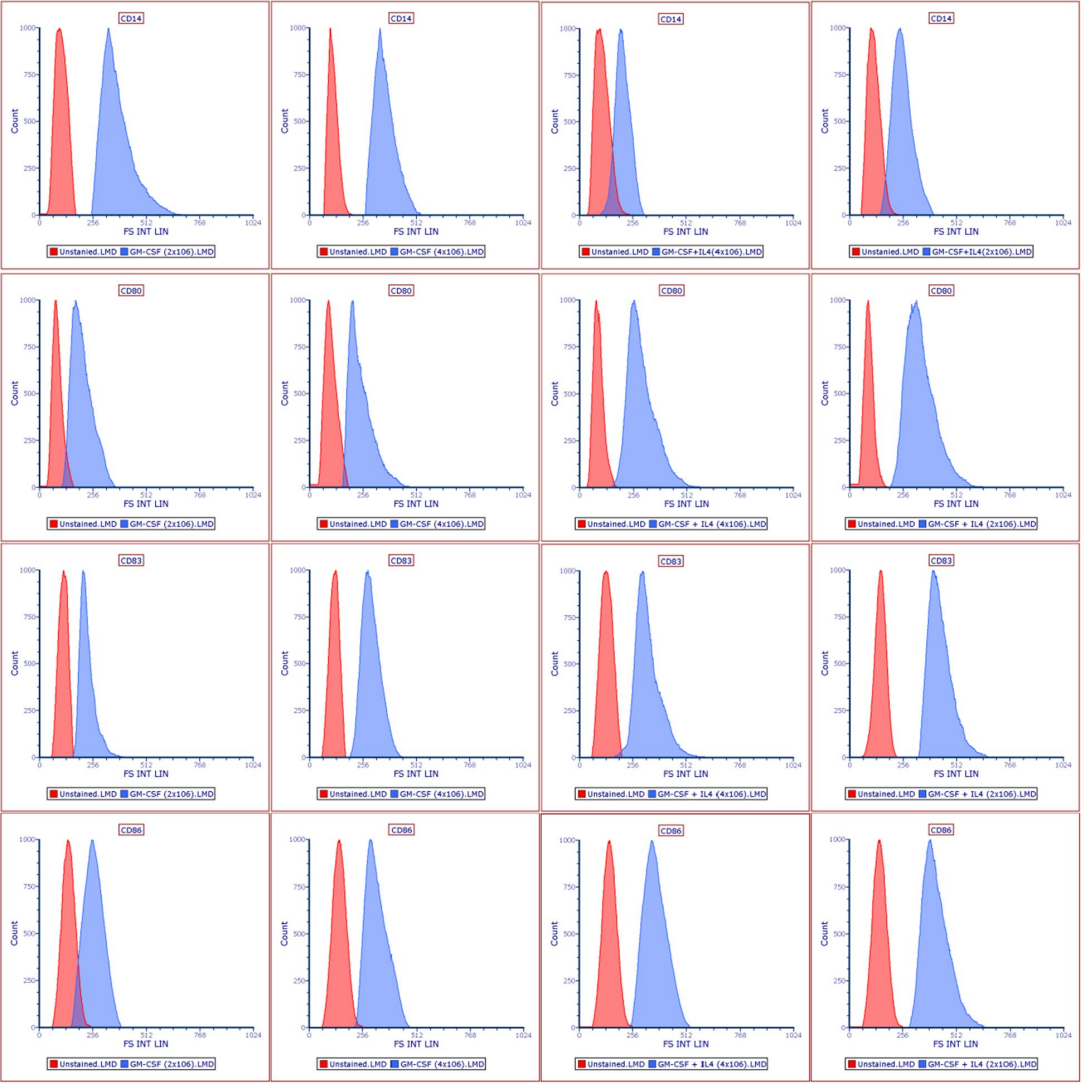
Representative histograms of BMDC surface marker expression. The histogram illustrates the role of combinations of two levels of the two variables individually based on the DC expression of surface markers. The highest fluorescence intensities of the mature BMDC markers (CD83, CD80, and CD86) were repeatedly detected in the cell group seeded at 2 × 10^6^ in the presence of GM-CSF and IL-4, followed by the cell group seeded at the higher density and treated with the cytokine mix

The morphological changes of mature BMDCs on day 7 were observed by using an inverted microscope (Table 3) (Figs. 2 and 3). Mature BMDCs were characterized by surface markers of high expression of CD80, CD86, and CD83, and low or lack of expression of monocyte markers CD14. The results showed that the lowest expression of CD14 was detected when both cytokines were used and with a cell density of 4 × 10^6^ cells/mL. CD83 expression was higher in the group of GM-CSF plus IL4 and with a density of 2 × 10^6^ cells/mL. Both CD80 and CD86 markers were also highly expressed in the group of GM-CSF plus IL4 and with lower cell density (i.e., 2 × 10^6^ cells/mL) compared to other sets of treatments (Table 3) (Figs. 1 and 4).

**Table 3:** Comparison of dendritic cell marker expression

	**GM-CSF & IL4**		**GM-CSF**		^*^P
	**2** × **10**^**6**^	**4** × **10**^**6**^	**2** × **10**^**6**^	**4** × **10**^**6**^	
**CD83 (MFI)**	157.6 ± 6.7	135.0 ± 4.9	76.1 ± 14.0	105.5 ± 4.9	0.049
**CD80 (MFI)**	316.2 ± 3.5	230.9 ± 2.3	131.8 ± 1.5	135.7 ± 2.1	0.012
**CD86 (MFI)**	390.6 ± 13.3	266.2 ± 8.6	186.5 ± 2.9	214.9 ± 5.0	0.001
**CD14 (MFI)**	172.8 ± 15.3	169.4 ± 19.5	282.9 ± 9.3	195.9 ± 18.6	0.03

*MFI* mean fluorescence intensity, *GM-CSF* granulocyte–macrophage colony-stimulating factor, *IL-4* interleukin-4

^*^P value was based on two-way ANOVA

### Optimization of experimental design

The design of the experiment (DoE) of the two variable levels was coded in (Table 1). Response values and the matrix of the factorial design are represented in (Table 4). The highest MFI was observed when media were supplemented with both GM-CSF and IL4 regardless of cell density. The factors of interest have shown significant influence on different estimated responses. To illustrate, the MFI of CD14 ranged from 169.4 to 282.9 arbitrary units (AU), while the MFI of co-stimulatory CD80 and CD86 was 131.8 to 316.7 AU and 186.5 to 390.6 AU, respectively. While CD83 maturation markers expressed at levels from 76.1 to 157.6 AU compared to the other markers. Therefore, the effect of the independent factors on marker expression (i.e., Y1, Y2 …) was illustrated by the factorial equation in (Table 5). Incorporating this information into the model, we can state that the calculated adjusted R2 coefficients, which surpass 0.8 in all models, provide robust evidence of the excellent alignment of the equations. This underscores that more than 80% of the variability in the response variable can be attributed to the fluctuations in the explanatory variables (A and B) within the model.

**Table 4:** The matrix of the experimental values and response values of BMDC marker expression conditions using (2^2^) factorial design

Run	Factor 1 (A)	Factor 2 (B)	Response 1	Response 2	Response 3	Response 4
	**Cytokine combinations (ng/mL)**	**Cell density (cells/mL)**	**CD14 expression (MFI AU)**	**CD83 expression (MFI AU)**	**CD80 expression (MFI AU)**	**CD86 expression (MFI AU)**
1	GM-CSF	2 × 10^6^	272.53	75.63	133.14	186.48
2	GM-CSF	2 × 10^6^	290.32	90.32	130.2	189.35
3	GM-CSF	2 × 10^6^	285.89	62.32	132.18	183.65
4	GM-CSF + IL4	2 × 10^6^	170.2	159.05	315.52	395.62
5	GM-CSF + IL4	2 × 10^6^	158.97	150.32	320.02	400.7
6	GM-CSF + IL4	2 × 10^6^	189.32	163.52	312.96	375.5
7	GM-CSF	4 × 10^6^	194.23	105.75	137.89	214.01
8	GM-CSF	4 × 10^6^	215.32	100.35	133.85	210.36
9	GM-CSF	4 × 10^6^	178.24	110.25	135.23	220.32
10	GM-CSF + IL4	4 × 10^6^	168.17	134.82	230.95	265.31
11	GM-CSF + IL4	4 × 10^6^	150.52	130.25	228.52	275.2
12	GM-CSF + IL4	4 × 10^6^	189.52	139.96	233.12	257.98

*MFI* mean fluorescence intensity

**Table 5:** The used linear regression equations to estimate the markers’ expression response

Response	Linear regression equation	*R*^2^	Adj.R^2^	^*^P
CD14	282.9 – 110.1 (A) – 86.9 (B) + 83.6 (AB)	0.924	0.895	< 0.001
CD83	76.1 + 81.5 (A) + 29.4 (B) – 51.9 (AB)	0.951	0.999	< 0.01
CD80	131.8 + 184.3 (A) + 3.8 (B) – 89.1 (AB)	0.999	0.989	< 0.001
CD86	186.5 + 204.1 (A) + 28.4 (B) – 152.8 (AB)	0.992	0.989	< 0.001

*Adj.R*^*2*^ adjusted *R*-squared

^*^P value was based on two-way ANOVA

### Main effects and interaction term

In terms of main effects for the CD83 marker, for instance, on average, one level increase in cytokine combination (i.e., GM-CSF and IL4) was associated with an 81.5 AU increase of CD83 MFI, keeping cell density constant (Table 5). The main effect of cell density was associated with only a 29.3 AU increase in CD83 AU for the group of 4 × 10^6^ cells/mL than the group of 2 × 10^6^ cells/mL. In a similar vein for CD14, using GM-CSF and IL4 cytokine combination corresponded to a decrease of 110.1 AU in MFI. Meanwhile, the main effect of cell density led to a reduction of 86.9 AU in MFI for the group at 4 × 10^6^ cells/mL compared to 2 × 10^6^ cells/mL. Shifting our focus to CD80, the main effect of cytokine combination was associated with a substantial increase of 184.3 AU in MFI, regardless of cell density. Conversely, the impact of cell density resulted in a modest 3.8 AU increase in CD80 MFI for the 4 × 10^6^ cells/mL group compared to the 2 × 10^6^ cells/mL group. Similarly, investigating CD86 revealed that the main effect of cytokine combination led to a noteworthy 204.1 AU increase in CD86 MFI, irrespective of cell density. On the other hand, cell density’s main effect contributed to a 28.4 AU elevation in CD86 MFI for the 4 × 10^6^ cells/mL group over the 2 × 10^6^ cells/mL group. These findings collectively underscore the differential impacts of cytokine combination and cell density on various markers, providing a comprehensive understanding of their influence on the maturation of dendritic cells.

The adjusted coefficient of *R*^2^ generated from the statistical analysis of models was 0.999, 0.989, and 0.989 for CD83, CD80, and CD86, respectively. The two variables and their interactions significantly contributed to the response of CD83 and CD86 (*P* < 0.001, two-way ANOVA). The maximum marker expression was associated with using GM-CSF and IL4 and lower cell density, MFI = 157.6 AU and MFI = 390.6 AU, for CD83 and CD86, respectively. That could be due to the sufficient number of cytokines, 20ng/mL and 10ng/mL of GM-CSF and IL4 can boost the maturation of 2 × 10^6^ cells/mL compared to 4 × 10^6^ cells/mL. On the other hand, only cytokines combination significantly contributed to the response of CD80 (*P* < 0.001, two-way ANOVA).

**Fig. 5:**
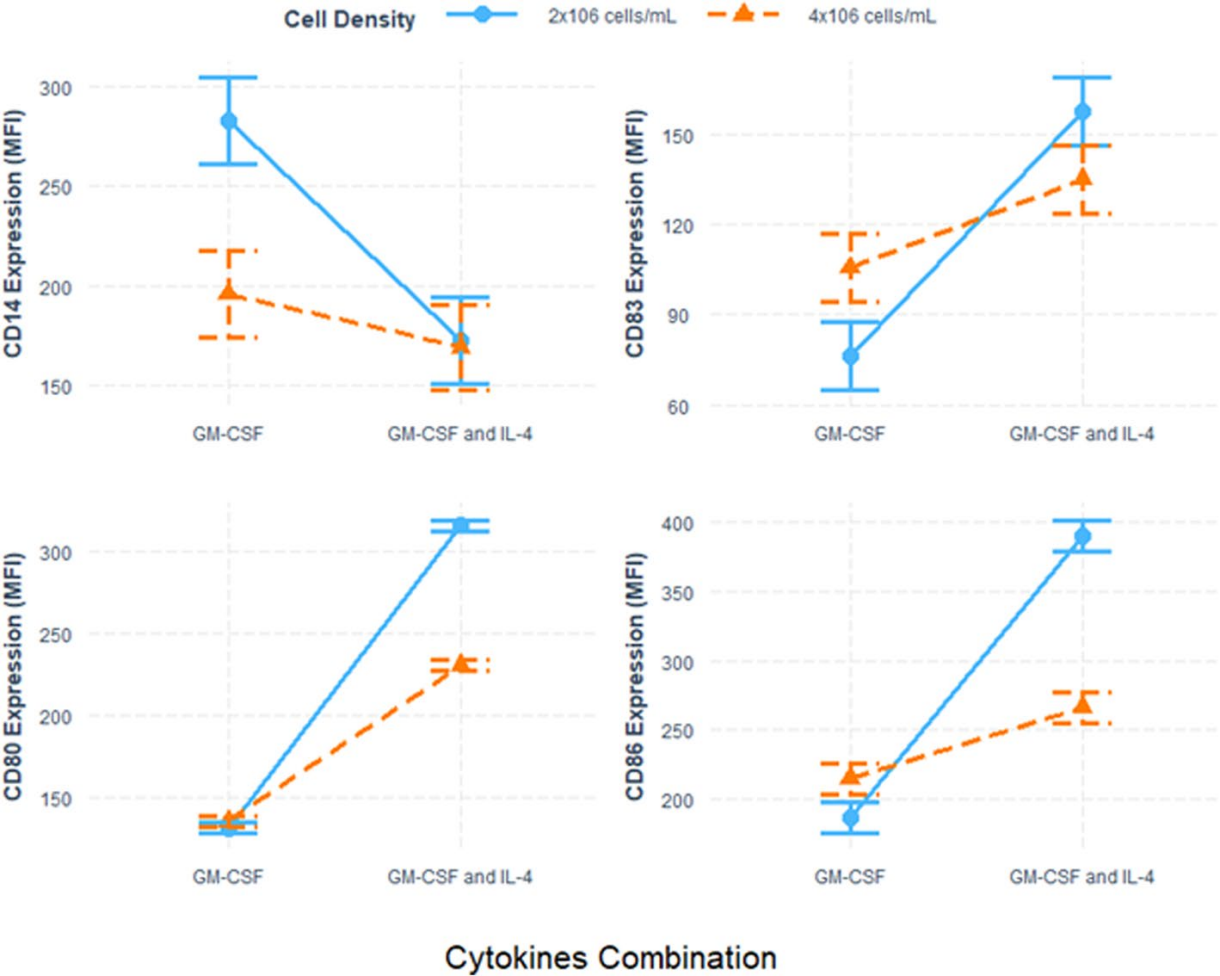
Two-factor interaction plot of the independent variables. There were significant interaction effects between cytokine combination and cell density on all DC markers (*P* < 0.001, two-way ANOVA)

Interestingly, the significant interaction between cytokine combination and cell densities across all four markers reveals the complex dynamics governing dendritic cell maturation (Table 5). The interaction plots provide a comprehensive picture of how these factors jointly influence the observed marker responses, underscoring the importance of considering their combined effects when optimizing dendritic cell culture conditions (Fig. 5). The magnitude of the positive interaction between cytokines combination and cell density is shown in Fig. 5 by non-parallel lines. That is, the effect of cell density of BMDC marker expression is different for different levels of cytokine combinations. For example, provided that low level of cytokine combination (i.e., GM-CSF), one level increase in cell density was associated with a 29.4 AU increase in CD83 marker expression compared to 22.5 AU when cells supplemented with GM-CSF and IL4. Indicating that higher cell density contributed to increasing the expression of BMDC markers if only GM-CSF was used. That might be due to the spontaneous maturation of using more than 2 × 10^6^ cells per mL.

## Discussion

Although factorial design has been recognized for its usefulness and importance in investigating interactions, several published protocols suggest a lack of systematic analysis into the effect of parameter levels and their interaction in culture performance. However, only a few experimental studies have used this design, such as cell-biomaterial [18, 19], optimization of culture conditions [20–22], tissue engineering [18, 23], and other biotechnological studies [24, 25]. Herein, applying factorial design in cell culture settings [26] has provided sufficient knowledge on optimizing the cell’s maturation.

In the current study, we systematically isolate and generate mature DCs from mice bone marrow under the aegis of GM-CSF and IL-4. For the generation of BMDCs, GM-CSF is essential to differentiate and the survival of DC progenitors and has proven to maximize pure DCs, according to [30–32]. Together, GM-CSF and IL-4 play complementary roles in the differentiation and activation of dendritic cells. Therefore, in the current study, the bone marrow of female BALB/c mice was used to obtain hematopoietic precursor cells, whereas non-DCs were removed by early washings [30, 31]. Morphological observations of unstimulated BMDCs showed a rounded shape with short or no projections (Fig. 2A, B). This observation aligns with the expectation that cytokine stimulation is necessary for inducing dendritic cell differentiation and maturation.

However, upon the addition of GM-CSF and IL4 at a cell density of 2 × 10^^6^ cells/mL, the DCs exhibited distinct morphological features (Figs. 2C and 3). This suggests that the combination of these cytokines at this particular density promoted dendritic cell maturation and the development of dendritic projections, which are essential for their immune-presenting function [10–12]. The distinct appearances of the mature BMDCs under different conditions shed light on the influence of cytokines and cell density on their morphology and maturation status (Fig. 2C–F).

BMDCs’ phenotypic characteristics were further investigated using flow cytometric analysis. This analysis is performed for different purposes; the most important reason is to accurately identify the desired subset of cells, especially when expressing the same surface markers such as DCs and macrophages [8, 17]. Therefore, we used four markers to identify DCs, including CD80, CD86, CD83, and CD14. Functional DCs activate T cells through two major signals: interaction between TCR and MHC complex and co-stimulatory signaling [2, 30, 31]. Unlike macrophages, mature DCs are characterized by high expression of co-stimulatory molecules, CD80, CD86, and membrane-bound CD83, and low expression or lack of monocyte markers CD14 [3].

The results of this work indicated that the cell yield, viability, and phenotypic markers’ expression are correlated with higher cytokine levels. Also, the effect of the maturation conditions and their interaction significantly contributed to the CD14 expression level (Y1) (*P* < 0.001, two-way ANOVA) (Table 4). Both cytokine combination and cell density were negatively correlated with CD14 expression level, the higher the level (i.e., two cytokines and higher cell density) the lower the monocyte CD14 expression (MFI = 169.4 AU) that indicates a successful differentiation of monocytes into DCs as reported by previous studies [3, 9]. Importantly, CD14 is a monocyte marker that occasionally decreases while monocytes differentiate into DCs [9].

Besides, the expression of the co-stimulatory molecules was significantly higher in DCs that were supplemented with both cytokines and seeded at 2 × 10^6^ cells/mL. However, CD86 was expressed at higher levels than CD80, as shown in (Table 2) that may be because CD86 expression on the APC surface is rapidly upregulated upon stimulation; CD80, on the other hand, requires stimulation to be expressed [33, 35]. Additionally, the membrane-bound CD83 is increased on the surface of activated DCs and is considered a major marker to distinguish between mature and immature DCs [36–38]. In general, the results of the expression of co-stimulatory molecules and mature DC markers were correlated with the low cell density.

Cytokine combinations have a positive effect on CD80 expression levels. The higher the level of cytokine combination, the higher the CD80 expression (MFI = 316.2 AU). Cell density has a difference of only 4.2 AU between DCs that are seeded in lower cell density compared to higher cell density. However, the higher MFI was correlated with a cell density of 2 × 10^6^ cells/mL and both cytokine combinations. To sum up, a cell density level of 2 × 10^6^ was positively correlated with BMDC marker expression when cells were supplemented with GM-CSF and IL4. Using only GM-CSF increased BMDC maturation with a cell density of 4 × 10^6^ cells/mL compared to IL4. Remarkably, the role of cytokines is strongly associated with BMDC maturation, even though all factors have significantly influenced BMDC maturation. Following the factorial approach, our optimization conditions ultimately resulted in an overall maturation for cells supplemented with GM-CSF and IL4 and seeded at a cell density of 2 × 10^6^ cells/mL.

## Conclusion

To our knowledge, the work presented here is one of a few investigations into the interaction effects of cell-culture variables using the DoE statistical approach. The maturation of DCs is implemented by culturing cells with the presence of cytokines. Then, they seeded at the desired number of cells per mL. Notably, because of the significant role of IL4 in DC maturation. Also, to avoid seeding more than 2 million cells per mL to eliminate spontaneous maturation. The current work has successfully optimized the DC maturation. In addition, it highlighted the importance of factorial experimental design in minimizing experiments, time, and process costs while maintaining high-quality responses.

## Data Availability

The datasets used and/or analyzed during the current study are available from the corresponding author on reasonable request.

## Abbreviations

DC: Dendritic cell
BMDC: Bone-marrow dendritic cells
DoE: Design of experiment
MHC: Major histocompatibility molecule
PAMP: Pathogen-associated molecular patterns
FACS: Flow cytometry staining buffer
FITC: Fluorescein isothiocyanate
GM-CSF: Granulocyte-macrophage colony-stimulating factor
IL4: Interleukin-4
AU: Arbitrary units
MFI: Mean fluorescence intensity
APC: Antigen-presenting cell
